# Ecologic Investigative Strategies to Determine Human Plague Exposure Sites, United States, 1991–2018

**DOI:** 10.3201/eid3204.251357

**Published:** 2026-04

**Authors:** Rebecca J. Eisen, Lynn M. Osikowicz, Erik Foster

**Affiliations:** Centers for Disease Control and Prevention, Fort Collins, Colorado, USA

**Keywords:** *Yersinia pestis*, plague, prevention, response, vector-borne infections, flea-borne, bacteria, bacterial infection, zoonoses, United States

## Abstract

Plague is a rare but potentially life-threatening fleaborne zoonotic disease caused by *Yersinia pestis*. Public health agencies in the United States use multiple concurrent epidemiologic and ecologic strategies to determine *Y. pestis* exposure sites. We reviewed 196 plague case files from 1991–2018 to describe effort and yield of implemented strategies. All files included an epidemiologic component, and 71% were followed up with environmental investigations. Environmental samples were collected for laboratory testing in 88% of investigations. The percentages of investigations yielding laboratory evidence of local transmission varied from 28% for testing live-trapped rodents to 50% for pet serology. We suggest that collection and laboratory testing of samples should be prioritized when epidemiologic investigations implicate potential exposure in an unusual setting, in areas where many people could be at risk of exposure to *Y. pestis,* or in situations where prevention activities extend beyond educational outreach and incur greater costs.

Plague is a rare but potentially life-threatening fleaborne zoonotic disease caused by the bacterium *Yersinia pestis*. Early diagnosis and effective antimicrobial treatment improve survival of plague infections (*1*). Awareness by the public and medical community of local *Y. pestis* transmission is critical to preventing plague cases and improving outcomes of infections (*2*,*3*). In the United States, plague is a nationally notifiable condition; healthcare providers and laboratories report plague cases to state and local health departments who then report confirmed and probable cases to the Centers for Disease Control and Prevention (CDC). Public health agencies promptly investigate cases and notify the public and healthcare providers of their occurrence. Along with those timely notifications, public health agencies encourage prevention measures (e.g., avoiding areas where plague epizootics have been detected, removing food and harborage for rodents in and around homes, avoiding handling sick or dead animals, and protecting people and pets from fleas), make recommendations on flea control or limiting access to public spaces undergoing active *Y. pestis* transmission, promote early medical care-seeking behavior if plague is suspected, and keep the medical community apprised of diagnosis and treatment guidelines (*2*,*4*–*7*). Accurately identifying when and where exposures to *Y. pestis* occur is central to effectively targeting this messaging.

In consultation or in collaboration with CDC, state and local public health departments follow up on human plague cases to determine where exposures to *Y. pestis* likely occurred and to identify ongoing risk. Place and mode of exposure are inferred from interviews with case patients or their close contacts on the basis of information provided about where the case-patient spent time before onset of symptoms, contact with sick or dead animals, or reports of flea or insect bites. To more definitively identify where *Y. pestis* is circulating in zoonotic hosts, environmental investigations are conducted at locations where the case-patient reportedly spent time within the expected incubation period. Such investigations range in complexity and quality of information gathered. Visual inspections of potential exposure sites are conducted to look for known risk factors (e.g., presence of food and harborage for rodents, evidence of rodent die-offs, and presence of free-roaming pets). Other strategies aim to collect specimens for laboratory testing to detect the presence of *Y. pestis* or provide serologic evidence of prior exposure to *Y. pestis* in pets, wildlife, or fleas (*2*,*8*–*10*). Such field methods include collecting blood from pets and information on any recent travel, searching for and collecting animal carcasses, swabbing animal burrows for host-seeking fleas, and live-trapping rodents and collecting their blood and infesting fleas. Finding laboratory evidence of *Y. pestis* at a potential exposure site increases confidence in the exposure site determination and is often beneficial when economically impactful decisions are made to prevent future cases (*4*).

To describe effort and yield of implemented ecologic strategies for plague, we reviewed CDC-maintained plague case files from 1991–2018. Our aim was to summarize when and where persons were exposed to *Y. pestis* in the United States, examine how often various investigative strategies were used to determine exposure sites, explore how often each of the methods aimed at collecting specimens for testing yielded laboratory evidence of current or prior *Y. pestis* transmission, and identify changes in public health practices related to exposure site determination.

## Methods

CDC maintains a list of human plague cases and supplemental information reported to the US Public Health Service (1900–1946) and CDC (1947–present). Epidemiologic trends derived from those files from 1900–2012 were reported previously (*6*). Common environmental exposures identified in case files from 1970–1991 have also been reported (*11*). We reviewed case files from 1991–2018 with an emphasis on the investigative strategies used to determine exposure locations. Although plague case reporting continued after 2018, we ended our review in 2018 to eliminate detecting time-limited changes in behavior or public health practices attributable to the coronavirus pandemic. At least 2 of the authors reviewed each case file to extract information on month of symptoms onset; state, county, and location of exposure (peridomestic or away from a home environment); and noted recreational or occupational exposures. Supplementary information on environmental investigations was not collected systematically. We noted whether each case file contained information on conducting site visits, carcass collections, burrow swabbing to collect host-seeking fleas, live-trapping of small mammals to collect blood samples and ectoparasites, and blood collection from pets for serologic testing. When plague-positive laboratory testing was noted in the files, we recorded which sample types tested positive. We described categorical data derived from case files as counts or percentages of case files. We used contingency table analyses to compare counts between categories (JMP Statistical Software, https://www.jmp.com). Animal procedures for CDC investigations from 2009 onward were reviewed and approved by the Institutional Animal Care and Use Committee of the CDC Division of Vector-Borne Diseases, National Center for Emerging and Zoonotic Infectious Diseases.

## Results

**Figure 1:**
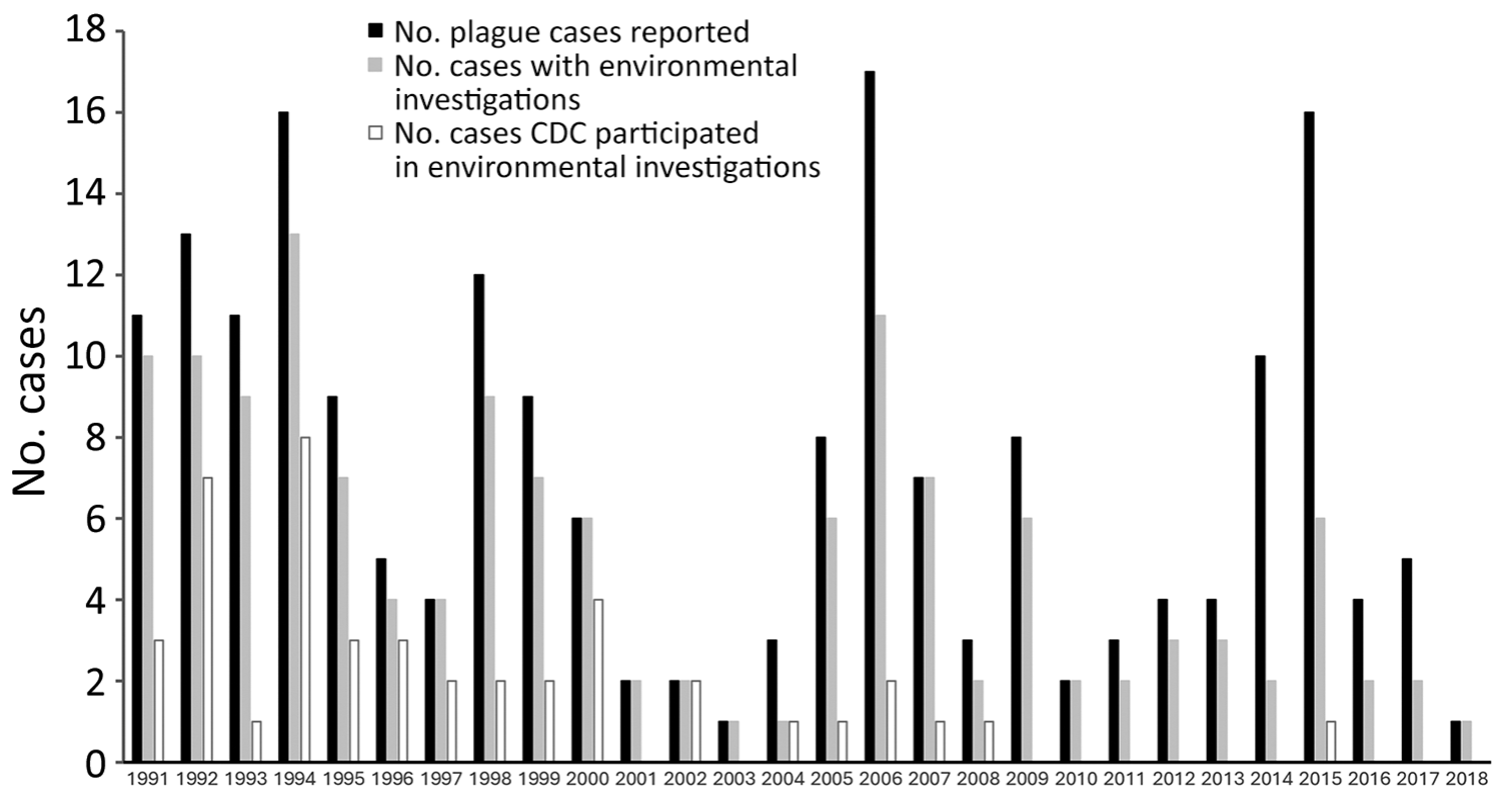
Reported plague cases and associated environmental investigations per year, United States, 1991–2018. The Centers of Disease Control and Prevention staff assisted state and local health departments in the field on a portion of case investigations.

During 1991–2018, a total of 196 human plague cases were reported to CDC. A median of 5.5 cases was reported per year, with as few as 1 case or as many as 17 cases reported in a year (Figure 1). Reported exposures occurred in 13 states: New Mexico (92 cases), Colorado (39 cases), Arizona (22 cases), California (14 cases), Oregon (9 cases), Utah (7 cases), Wyoming (3 cases), Idaho (2 cases), Nevada (2 cases), Oklahoma (2 cases), Illinois (1 laboratory acquired infection), Montana (1 case), and Texas (1 case); the state of exposure was not determined for 1 case. Most case-patients (85%) were reportedly exposed to *Y. pestis* in southwestern states, including New Mexico (47%), Colorado (19%), and Arizona (11%), or in California (7%). Onset of symptoms was reported in April–October for 91% (n = 179) of cases. Most exposures were determined to have occurred in and around a home (peridomestic exposures) (68%, n = 133). Of the 47 (24%) case-patients exposed to *Y. pestis* away from a home setting, 36 were associated with recreational activities (e.g., camping, hiking, jogging, or hunting), and 10 were considered occupational exposures (e.g., veterinary medical professionals [n = 4], wildlife biologists and trappers [n = 5], or laboratorian [n = 1]). Subcounty level exposure sites were not determined for 16 (8%) cases. Most cases reported from New Mexico (74 cases, 80%), Colorado (28 cases, 71%), and Arizona (13 cases, 59%) were categorized as peridomestic exposures. In contrast, only 36% of California cases (5 cases) were categorized as peridomestic exposure; 50% (7 cases) were exposed away from the home, and exposure location was not determined for 14% (2 cases) of California cases.

State and local health departments conducted environmental investigations to determine exposure sites and to assess ongoing risk of exposure to *Y. pestis*. Findings of environmental investigations were documented in 140 case files (71%); CDC assisted in the field with 44 (31%) of these investigations (Figure 1). We noted a sharp decline in environmental investigations conducted in the most recent decade (χ^2^ = 15.85; p<0.001) (Figure 1). Cumulatively from 1991–2008, environmental investigations were conducted for 80% (111) of 139 cases. By contrast, from 2009–2018, environmental investigations were conducted for only 51% of cases (29 of 57 cases). CDC’s involvement in field activities declined significantly in the most recent decade compared with the prior decades (χ^2^ = 18.05; p<0.001) (Figure 1). CDC assisted state and local health departments in the field with 39% (44) of 111 environmental investigations during 1991–2008, compared with assisting with only 1 of 29 environmental investigations during 2009–2018.

No environmental investigations were recorded for 56 (29%) of 196 cases. Among the 140 (71%) case files with accompanying information on environmental investigations, 136 (97%) reported visiting potential exposure sites and conducting observational risk assessments (e.g., looking for signs of rodent activity/inactivity, availability of food and harborage for rodents). The 4 cases lacking information on site visits were associated with animal exposures in which case files indicated animals or animal carcasses were tested, but no additional information on site observations was provided. Among the observational studies conducted in 136 cases, 51 (38%) noted signs of rodent die-offs.

**Figure 2:**
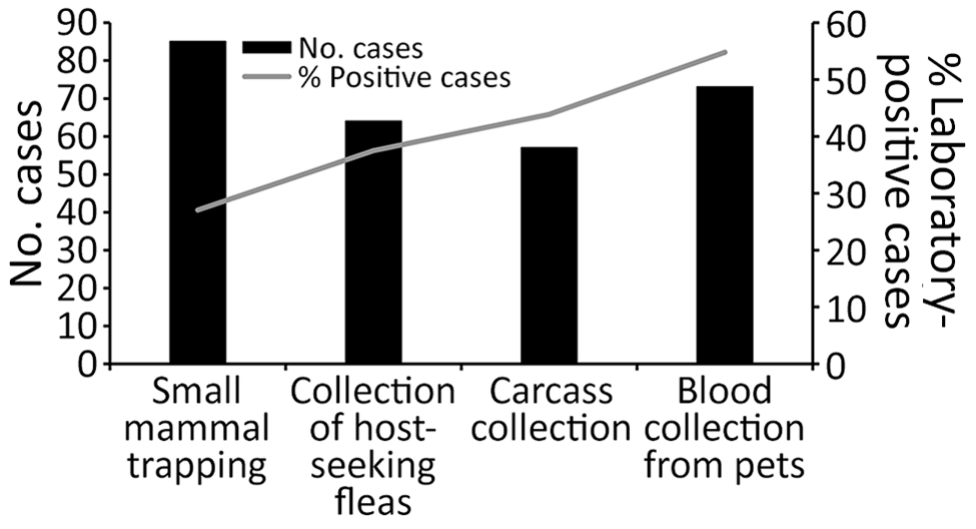
Numbers of plague case files (n = 140) that contained records of environmental investigations and noted using methods intended to collect laboratory testing samples, United States, 1991–2018. Methods included small mammal trapping (n = 85 cases), collection of pet serum (n = 73 cases), burrow swabbing to collect host-seeking fleas (n = 64 cases), or carcass collection (n = 57 cases). Percentages of cases where laboratory evidence of *Y. pestis* transmission was recorded in case files are shown for each sample acquisition method. Visual assessments were conducted for 136 cases (data not shown).

A total of 123 (63%) of 196 case files (88% of 140 case files noting environmental investigations) indicated use of >1 method to obtain samples for laboratory testing (e.g., small mammal trapping to collect blood and flea samples, burrow swabbing to collect host-seeking fleas, carcass collection, or blood collection from pets) (Figure 2). Plague positive samples were detected for 82 (67%) of 123 cases for which methods were used to collect samples for laboratory testing. A total of 85 (61%) of 140 case files with investigations included evidence of live-trapping of small mammals; among those, rodents or their infesting fleas tested positive for *Y. pestis* in 24 (28%) of the case investigations. Seventy-four (53%) case files indicated blood was collected from pets (cats or dogs) for serologic testing; among those, 37 (50%) investigations yielded seropositive results. Attempts to collect host-seeking fleas from rodent burrows were noted in 64 (46%) case files; among them, 24 (38%) investigations yielded plague positive fleas. Fifty-seven (41%) case files reported attempts to collect animal carcasses, of which 27 (47%) yielded laboratory evidence of *Y. pestis*. We believe this report is likely an underestimate of the actual effort to find carcasses because the notation of carcass searches was not systematic. When carcasses were found, they were noted in the files. However, a few instances reported looking for but not finding carcasses.

Compared with other methods that yield samples for laboratory testing (burrow swabbing, carcass collection, or collection of blood from pets), live-trapping small mammals and collecting their blood and infesting fleas and testing the resulting samples is the most time consuming and financially costly. We sought to determine the yield from those efforts. Most environmental investigations used multiple methods to collect environmental samples for testing (59%, 83 of 140 case files). Among the 24 cases where blood or fleas derived from live-trapping yielded plague positive laboratory results (17 case files with seropositive rodents, 10 with *Y. pestis* detected in infesting fleas; 3 case files in which both sample types were positive), 16 (67%) yielded positive laboratory results from other methods as well (e.g., pet serum, carcasses, or fleas collected from burrows). Only 8 environmental investigations (<10% of 82 cases with laboratory evidence for determining exposure sites) provided laboratory support for local transmission of *Y. pestis* based solely on live-trapping (i.e., other environmental samples did not yield positive laboratory testing results).

Fifty case files included laboratory evidence implicating various wildlife taxa in local *Y. pestis* transmission. Laboratory evidence included direct detection or serologic detection of *Y. pestis* in live trapped rodents or direct detection in infesting or host-seeking fleas, or direct detection of *Y. pestis* from animal carcasses. Most files (80%, n = 40 files) identified a single taxon exposed to *Y. pestis*; 9 files identified *Y. pestis* in 2 taxa and 1 file identified 3 infected taxa. Ground squirrels (40%, n = 20 files) and prairie dogs (24%, n = 12) were the most common taxa implicated in local transmission. Other infected taxa included *Peromyscus* mice (18%, n = 9 files), woodrats (16%, n = 8 files), rabbits (12%, n = 6 files), chipmunks or bobcats (4%, n = 2 files), and a mountain lion and a tree squirrel (2%, n = 1 file).

## Discussion

Most (71%) human plague cases reported for 1991–2018 were followed up with environmental investigations to determine exposure locations. However, we found a major decline in environmental investigations in the most recent decade evaluated. In that decade, exposure sites for plague cases were mostly inferred solely on the basis of case histories. When environmental investigations were conducted, observational risk assessments were almost always conducted. Nearly two thirds of case files also contained information indicating that methods were used to collect environmental samples for laboratory testing; among those cases, two thirds of field investigations yielded laboratory support for recent or ongoing *Y. pestis* transmission at the site investigated. Determinations of exposure sites were supported by laboratory evidence of *Y. pestis* in environmental samples in 42% of 196 reviewed case files.

Each investigative strategy has advantages and challenges. To overcome those challenges, multiple methods were often deployed within each environmental investigation to obtain environmental samples for testing. Small mammal trapping was the most noted method in case files, followed by blood collection from pets, collection of host-seeking fleas from rodent burrows, and collection of carcasses. Of note, those are rough estimates that depict broad trends in effort and yield. Notation of environmental investigation data within case files was not systematic or complete. Nonetheless, our review indicated that live-trapping was the method least likely to yield laboratory evidence of recent (rodent serology) or ongoing (direct detection in infesting fleas) *Y. pestis* transmission; only 28% of cases including live-trapping yielded laboratory evidence of *Y. pestis*. By contrast, nearly half of case files that included either pet serologic testing or direct detection of *Y. pestis* in collected carcasses yielded laboratory support for recent local transmission. Live-trapping of rodents is the most labor-intensive and costly strategy used in environmental investigations, and it rarely provides unique support for local plague transmission. In situations where plague activity was detected in live-trapped rodents or their infesting fleas, carcasses, host-seeking fleas, or pet serum often provided additional evidence of *Y. pestis* transmission. Because of the relatively low yield and more labor-intensive effort of live-trapping, the method might only be beneficial in unusual outbreaks.

Most human plague cases reported in the United States are associated with epizootic activity (*2*,*6*,*11*,*12*). During plague epizootics, fleaborne *Y. pestis* spreads rapidly among susceptible mammalian hosts, often ground squirrels and prairie dogs. As large numbers of rodents die from plague infection, their associated fleas seek alternative bloodmeal sources, including feeding on humans and their pets (*2*,*13*–*15*). Carnivores, lagomorphs, and other mammalian hosts are often exposed to *Y. pestis*, particularly during epizootic periods, through direct contact with infected rodents or exposure to their infectious fleas. Susceptibility among nonrodent hosts varies widely (*14*,*15*). The high mortality rate among rodents and other mammalian hosts explains the high success rate in detecting *Y. pestis* in carcasses found during case investigations. Although carcass testing yielded a high percentage of positive samples, finding carcasses can be difficult in some settings where scavengers remove carcasses or where carcasses are obstructed by vegetation, burrows, or other obstacles (*8*,*16*,*17*). This difficulty in finding carcasses is reflected in the low percentage of investigations that collect carcasses. Because carnivores and scavengers are exposed to rodents and their fleas over large areas, serologic testing of wild and domestic carnivores has proven to be effective in detection of prior exposures to *Y. pestis* in animals that survive infections (*18*–*22*). Monitoring seroprevalence in carnivores and scavengers is more commonly implemented as a surveillance strategy, rather than response. Some rodent species show a heterogeneous response to infection; that is, some rodents die from infection, but others survive and seroconvert (*13*,*14*). Live-trapping capitalizes on detecting the small percentage of animals that were exposed to *Y. pestis*, survived infection, and seroconverted (*4*,*23*,*24*). Direct detection of *Y. pestis* in rodent blood or tissues is not conducted routinely because active infections in susceptible rodents are often fatal, reducing the odds of capturing a live infected rodent (*13*,*25*). Success in detecting plague activity in live-trapped rodent samples is dependent on the timing and location of trapping relative to the spread of *Y. pestis* in rodent communities, trapping effort, the composition of the rodent community, and the susceptibility or resistance to *Y. pestis* of species captured also influences the likelihood of capturing seropositive animals (*4*,*16*,*23*,*24*,*26*). Collecting and testing host-seeking fleas is often useful in detecting *Y. pestis* in fleas that have fled their dead or dying hosts, but success is dependent upon detecting a recent die-off and finding rodent burrows in the affected area. Although prevalence of *Y. pestis* in fleas is elevated during epizootics, even during active epizootics, prevalence of *Y. pestis* infection in fleas is often low (*23*,*26*,*27*).

Although fleaborne transmission remains the most common mode of *Y. pestis* transmission to humans, direct contact with infectious animals or animal carcasses is also a major mode of animal-to-human transmission (*2*,*6*,*11*,*28*–*30*). In recent decades, case investigations revealed that most plague case-patients were exposed to *Y. pestis* in the peridomestic environment in rural or semi-rural parts of the southwestern United States (*5*,*6*,*29*,*31*), where availability of food and harborage for rodents in the home environment is a known risk factor (*29*,*32*). Such food resources increase the chances of bringing potentially infectious rodents and their fleas into the home environment where they pose a risk to humans directly (flea bites, direct contact with infected animal carcasses) or indirectly (exposure to sick pets that were exposed to *Y. pestis* in and around the home). Cats are acutely susceptible to plague, and bites, scratches, or exposure to respiratory droplets of plague-infected cats pose a major hazard to humans. Veterinary staff and care takers treating sick cats are at elevated risk of plague infection (*28*,*33*,*34*). Although dogs exposed to *Y. pestis* can show clinical signs of plague infection, illness is often less severe in dogs compared with cats (*33*). Nonetheless, plague-infected dogs are implicated in direct transmission to humans (*35*,*36*). More often, pet dogs serve as phoretic hosts, bringing potentially infectious fleas into the home environment and sleeping in the same bed as a dog was identified as a major risk factor for plague (*37*,*38*). Humans spend a considerable amount of time in the same risk environments as their pets, but pets are usually more likely to come into direct contact with infected rodents and their fleas. As a result, pet serology is an effective means of identifying potential exposure sites in case investigations. However, pets (particularly dogs) often travel with humans and therefore collecting travel histories for the pets is essential to discerning exposure locations on the basis of pet serology alone.

The percentage of cases that led to public health agencies conducting environmental investigations declined sharply in the most recent decade evaluated. The reasons for this decline are not entirely clear, but we believe it coincided with changes in the World Health Organization’s International Health Regulations (IHR; https://apps.who.int/gb/bd/pdf_files/IHR_2014-2022-2024-en.pdf), staffing at public health agencies, and competing priorities including responding to more common emerging vectorborne disease threats. Under the previous version of the IHR, published in 1969, all human plague cases required reporting to the World Health Organization. Under the updated IHR (2005), implemented in 2007, notification to the World Health Organization is required only for unexpected events that present a risk for international spread (*39*). After decades of conducting epidemiologic and environmental investigations of plague cases in the United States, geographic and behavioral risk factors are well characterized (*2*,*5*,*6*,*11*,*25*,*31*). Because of that knowledge, usual exposures in western states associated with outdoor activities, noted flea bites, or direct contact with sick or dead animals can be inferred through case histories alone.

Detecting *Y. pestis* in environmental samples builds confidence in determining locations of exposure and assessing ongoing transmission risk. Use of multiple concurrent environmental investigative strategies has proven effective in providing laboratory evidence of local *Y. pestis* transmission; however, the individual components have associated costs. It is worth scrutinizing the precision and accuracy of environmental data needed to inform public health action relative to the resources expended to obtain supportive environmental data. Most often, prevention focuses on educating the public and healthcare community of plague risk and prevention strategies. The exact location and extent of epizootic transmission might not be needed for such outreach, unless a plague case occurs in a densely populated setting. In instances where many people are at risk for exposure to *Y. pestis*, public health entities might recommend vector control or limiting site access (*4*,*16*). Laboratory evidence of ongoing transmission provides support for those decisions. Moreover, environmental sampling provides baseline data that can be used to assess the effectiveness of vector control efforts. We suggest that environmental investigations, particularly collection of samples for laboratory testing, should be prioritized when epidemiologic investigations indicate potential exposure in an unusual setting, in areas where large populations could be at risk for exposure to *Y. pestis*, or in situations where prevention activities extend beyond educational outreach and incur greater costs.
